# CNP regulates cardiac contractility and increases cGMP near both SERCA and TnI: difference from BNP visualized by targeted cGMP biosensors

**DOI:** 10.1093/cvr/cvab167

**Published:** 2021-05-10

**Authors:** Ornella Manfra, Gaia Calamera, Alexander Froese, Dulasi Arunthavarajah, Nicoletta C Surdo, Silja Meier, Arne Olav Melleby, Monica Aasrum, Jan Magnus Aronsen, Viacheslav O Nikolaev, Manuela Zaccolo, Lise Román Moltzau, Finn Olav Levy, Kjetil Wessel Andressen

**Affiliations:** Department of Pharmacology, Institute of Clinical Medicine, University of Oslo and Oslo University Hospital, P.O. Box 1057 Blindern, 0316 Oslo, Norway; Department of Pharmacology, Institute of Clinical Medicine, University of Oslo and Oslo University Hospital, P.O. Box 1057 Blindern, 0316 Oslo, Norway; German Center for Cardiovascular Research, University Medical Center Hamburg-Eppendorf and Institute of Experimental Cardiovascular Research, Hamburg, Germany; Department of Pharmacology, Institute of Clinical Medicine, University of Oslo and Oslo University Hospital, P.O. Box 1057 Blindern, 0316 Oslo, Norway; Department of Physiology, Anatomy and Genetics, Oxford University, Oxford, UK; Department of Pharmacology, Institute of Clinical Medicine, University of Oslo and Oslo University Hospital, P.O. Box 1057 Blindern, 0316 Oslo, Norway; Department of Molecular Medicine, Institute of Basic Medical Sciences, University of Oslo, Oslo, Norway; Department of Pharmacology, Institute of Clinical Medicine, University of Oslo and Oslo University Hospital, P.O. Box 1057 Blindern, 0316 Oslo, Norway; Department of Pharmacology, Institute of Clinical Medicine, University of Oslo and Oslo University Hospital, P.O. Box 1057 Blindern, 0316 Oslo, Norway; Department of Molecular Medicine, Institute of Basic Medical Sciences, University of Oslo, Oslo, Norway; German Center for Cardiovascular Research, University Medical Center Hamburg-Eppendorf and Institute of Experimental Cardiovascular Research, Hamburg, Germany; Department of Physiology, Anatomy and Genetics, Oxford University, Oxford, UK; Department of Pharmacology, Institute of Clinical Medicine, University of Oslo and Oslo University Hospital, P.O. Box 1057 Blindern, 0316 Oslo, Norway; Department of Pharmacology, Institute of Clinical Medicine, University of Oslo and Oslo University Hospital, P.O. Box 1057 Blindern, 0316 Oslo, Norway; Department of Pharmacology, Institute of Clinical Medicine, University of Oslo and Oslo University Hospital, P.O. Box 1057 Blindern, 0316 Oslo, Norway

**Keywords:** NPR, Natriuretic peptide receptor, AKAP18δ, Biosensor, PLN

## Abstract

**Aims:**

Guanylyl cyclase-B (GC-B; natriuretic peptide receptor-B, NPR-B) stimulation by C-type natriuretic peptide (CNP) increases cGMP and causes a lusitropic and negative inotropic response in adult myocardium. These effects are not mimicked by NPR-A (GC-A) stimulation by brain natriuretic peptide (BNP), despite similar cGMP increase. More refined methods are needed to better understand the mechanisms of the differential cGMP signalling and compartmentation. The aim of this work was to measure cGMP near proteins involved in regulating contractility to understand compartmentation of cGMP signalling in adult cardiomyocytes.

**Methods and results:**

We constructed several fluorescence resonance energy transfer (FRET)-based biosensors for cGMP subcellularly targeted to phospholamban (PLB) and troponin I (TnI). CNP stimulation of adult rat cardiomyocytes increased cGMP near PLB and TnI, whereas BNP stimulation increased cGMP near PLB, but not TnI. The phosphodiesterases PDE2 and PDE3 constrained cGMP in both compartments. Local receptor stimulation aided by scanning ion conductance microscopy (SICM) combined with FRET revealed that CNP stimulation both in the t-tubules and on the cell crest increases cGMP similarly near both TnI and PLB. In ventricular strips, CNP stimulation, but not BNP, induced a lusitropic response, enhanced by inhibition of either PDE2 or PDE3, and a negative inotropic response. In cardiomyocytes from heart failure rats, CNP increased cGMP near PLB and TnI more pronounced than in cells from sham-operated animals.

**Conclusion:**

These targeted biosensors demonstrate that CNP, but not BNP, increases cGMP near TnI in addition to PLB, explaining how CNP, but not BNP, is able to induce lusitropic and negative inotropic responses.

## Graphical Abstract

**Figure:**
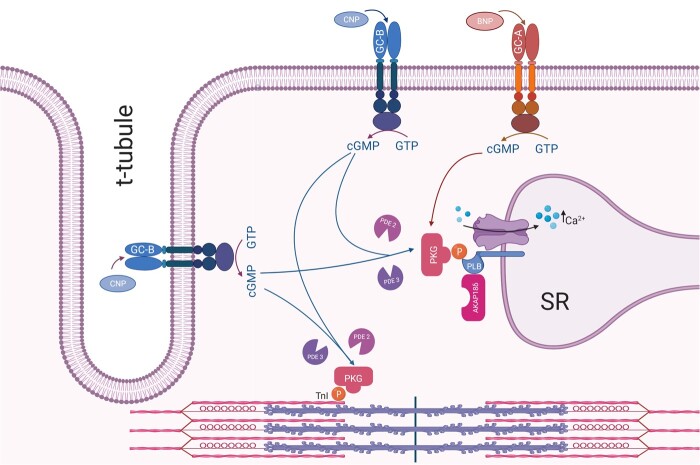

## 1. Introduction

The cyclic nucleotide 3’,5’-cyclic guanosine monophosphate (cGMP) is an intracellular second messenger with important effects in the cardiovascular system. In the heart, cGMP synthesis is catalysed by three types of guanylyl cyclases (GCs): one soluble cytosolic (sGC) activated by nitric oxide and the two plasma membrane bound natriuretic peptide (NP) receptors A and B (NPR-A and -B, now termed GC-A and GC-B), where GC-A is activated by atrial (ANP) and brain NP (BNP), and GC-B by C-type NP (CNP).^1^^,^^2^ The subcellular localization, duration, and amplitude of cGMP signalling are regulated not only by its production, but also by its degradation by different phosphodiesterases (PDEs).^3^ In the heart, PDE2, 3, 5, and 9 are the main cGMP-degrading PDEs.^4^^,^^5^

In heart failure, treatment with an angiotensin receptor–neprilysin inhibitor, elevating natriuretic peptide levels, improved heart failure with reduced ejection fraction (HFrEF)^6^ and, in women, also heart failure with preserved ejection fraction (HFpEF).^7^ Also in heart failure, myocardial CNP levels are increased^8^^,^^9^ and CNP increases cGMP production in cardiomyocytes in rodent models.^10–14^ In addition, CNP stimulation was recently shown to reduce myocyte stiffness.^15^^,^^16^ We previously reported that, in cardiomyocytes from failing hearts, CNP activates GC-B, resulting in cGMP-mediated phosphorylation of phospholamban (PLB) and troponin I (TnI).^11^^,^^17^ This results in increased Ca^2+^ sequestration by the SR [via increased sarcoplasmic reticulum Ca^2+^ ATPase 2 (SERCA2) activity] and decreased myofilament Ca^2+^ sensitivity, causing a lusitropic (LR) and negative inotropic response (NIR).^11^^,^^17–19^ In addition, we have also shown that CNP enhances the cAMP-mediated inotropic response of β_1_-adrenergic and 5-HT_4_ serotonin receptor stimulation through cGMP-dependent PDE3 inhibition.^11^^,^^20^ It is puzzling that neither of these effects were observed when activating the GC-A receptor with BNP despite comparable increase in total cGMP levels.^10^^,^^11^^,^^13^ One interpretation is that GC-B, but not GC-A, increases cGMP in subcellular compartments that yield PLB and TnI phosphorylation. However, tools to test this hypothesis have been lacking. In this study, we therefore wanted to elucidate the compartmentation of cGMP following GC-A and GC-B stimulation by monitoring cGMP levels using localized biosensors.

The development of sensitive and selective fluorescence resonance energy transfer (FRET)-based biosensors allows for monitoring of intracellular cGMP in intact living cells. Such biosensors contain a cGMP-binding domain that changes conformation upon cGMP binding, sandwiched between two fluorescent proteins, the relative position of which determines FRET.^21^ In this study, we used two different FRET-based biosensors: cygnet2.1 and red-cGES-DE5, which display different sensitivity for cGMP (µM- and nM-range, respectively).^22^^,^^23^ These biosensors were targeted to either PLB, through A-kinase anchoring protein 18δ (AKAP18δ) that interacts with PLB and SERCA2,^24^ or TnI, to relate changes in cGMP levels near these proteins to functional changes upon stimulation of GC-A and GC-B. In addition, we used scanning ion conductance microscopy (SICM) combined with FRET^25^ to determine whether receptors in t-tubules or at the cell crest could mediate these responses. Finally, we examined whether some of these effects were modified in heart failure.

## 2. Methods

### 2.1 Materials

Cilostamide (Cil 1 μM) and sildenafil (Sfil 100 nM) were from Tocris Bioscience (Bristol, UK). C-type natriuretic peptide (CNP 300 nM) and brain natriuretic peptide (BNP 300 nM) were from GenScript Corp (Piscataway, NJ) and Bachem (Bubendorf, CH). Antibodies against pTnI (Ser23/Ser24), total TnI, and vinculin were from Cell Signaling (Beverly, MA), and antibody against pPLB (Ser16) and total PLB were from Badrilla (Leeds, UK). All other chemicals and medium, unless otherwise noted, were from Sigma-Aldrich (Sigma-Aldrich, St. Louis, MO).

### 2.2 Development of targeted biosensors

Plasmids: cygnet2.1 biosensor was previously described,^26^ AKAP18δ construct was a kind gift from Enno Klussmann, Max Delbrück Center, Berlin, and TnI was provided by George Baillie, University of Glasgow, UK. The red-cGES-DE5 biosensor^23^ N-terminally flanked with *Xba*I and a linker A(EAAAK)^5^A to separate the domains of the fusion proteins and keep their full flexibility^27^ were synthesized and inserted into a pUC57 vector by Genscript and transferred to pcDNA3.1. The TnI-red-cGES-DE5 and AKAP18δ-red-cGES-DE5 biosensors were constructed by inserting *Nhe*I/*Xba*I inserts into *Nhe*I/*Xba*I opened red-cGES-DE5 biosensor. The corresponding cygnet2.1 biosensors were constructed by inserting *Xho*I/*Eco*RI cut cygnet2.1 to replace red-cGES-DE5. Cygnet2.1, Red-cGES-DE5, TnI-red-cGES-DE5, and AKAP18δ-red-cGES-DE5 were packaged in adenovirus, amplified, and titre measured by VectorBiolabs (Malvern, PA).

### 2.3 Animals

This investigation was approved by the Norwegian Food Safety Authority (application number 4938, 6581, 8683, and 23193) and conforms to the research animal Directive 2010/63/EU, the Guide for the Care and Use of Laboratory Animals (NIH publication No. 85-23, revised 2011, USA), in accordance with the ARRIVE guidelines.^28^ Animals were anaesthetized with isoflurane, and anaesthesia was confirmed by abolished pain reflexes. Animals were euthanized by cervical dislocation, and the chest was then opened to extract the heart.

### 2.4 Aorta banding in rats

O-rings of rubber with internal diameter of 1.2 mm (Apple rubber, USA) were prepared by opening of the ring and placing a non-absorbable 5–0 suture through both ends of the opening cut (see Melleby *et al.* for illustration of preparation technique).^29^ Male Sprague Dawley rats (∼120-140 g; Janvier, France) were anaesthetized in an anaesthesia chamber with 5% isoflurane and 95% oxygen, before endotracheal intubation and ventilation using a VentElite Small Animal Ventilator (Harvard Apparatus, USA). Anaesthesia was maintained with a combination of 2% isoflurane and 98% oxygen. Animals were placed in a supine position at a heating pad and hair removed in the incision area. A lateral, longitudinal incision was made at the right side of the thorax, and the pectoral muscles were separated using blunt scissors and retracted. The third or second intercostal muscle was pierced, and the ascending aorta was accessed after retraction of the costae and thymus. Periaortic fat was trimmed, and the pre-prepared O-ring was placed around the ascending aorta proximal to the brachiocephalic trunk. The sutures in the O-ring were tightened, the retractors were removed, and the skin was sutured using 5-0 silk sutures. The lungs were inflated to avoid pneumothorax, and the animals were ventilated with 100% oxygen until spontaneous ventilation. Sham-operated rats were subjected to a similar procedure, but without placement of an O-ring. Analgesia was maintained with pre-surgical followed by repetitive post-operative subcutaneous injections of buprenorphine 0.1 mg/kg 1–3x per day in the five days following surgery. All animals were followed with at least daily inspection, and stored in cages with three rats per cage and *ad libitum* supply of food and water under 12/12 h light/dark cycles.

### 2.5 Echocardiography

Echocardiography was performed 6 weeks after aorta banding. Rats were anesthetized in an anaesthesia chamber with a mixture of 5% isoflurane and 95% oxygen, and maintained by mask ventilation using a mixture of 1.5% isoflurane and 98.5% oxygen. Echocardiographic measurement of left ventricular and atrial dimensions in systole and diastole was performed and blinded to animal groups, and Doppler images of mitral, pulmonary, and aortic blood flow were performed in addition to recordings of tissue velocities of the posterior wall of the left ventricle in long axis. Heart and lung weights were measured after euthanasia.

### 2.6 Isolation of cardiomyocytes

Cardiac ventricular myocytes from 1- to 3-day-old Sprague Dawley rats (Charles River Laboratories, Wilmington, MA) were prepared as previously described^30^ and transfected with TransFectin Lipid Reagent (BioRAD, Hercules, CA) according to the manufacturer’s protocol. Adult left-ventricular cardiomyocytes were isolated from hearts from adult Wistar rats and Sprague Dawley rats from aorta banding by perfusion (Langendorff set-up) with a Ca^2+^ free Joklik-MEM buffer and digested enzymatically by using collagenase type II (90 U·mL^−1^) (Worthington Biochemical Corp., Freehold, NJ, USA) and prepared as previously described.^31^

### 2.7 Transfection of HEK293T cells and in vitro assay

HEK293T cells were cultured in Dulbecco’s modified Eagle’s medium (Invitrogen, Life technologies, Carlsbad, CA) with 10% foetal bovine serum, penicillin (100 U/ml), and streptomycin (100 μg/ml). Cells were transiently transfected using LipofectAMINE 2000 (Invitrogen) according to the manufacturer`s protocol, with the indicated plasmids. After transfection, cells were cultured in UltraCULTURE (Lonza, Basel, Switzerland), supplemented with L-glutamine (2 mM), penicillin (100 U/ml), and streptomycin (100 μg/ml) for 48 h. Cells were then homogenized with Ultraturrax (IKA, Staufen, DE) for 30 sec at 4°C in Buffer A (mM): HEPES (10) pH 7.3, NaCl (137), KCl (5.4), CaCl_2_ (2), MgCl_2_ (1), and homogenates were stimulated with increasing concentrations of cGMP in the presence of IBMX (100 μM). The donor fluorophores CFP and T-SapphireΔC11 were excited at 430 nm or 405 nm, respectively, and the fluorescent emission (F) of citrine/CFP (515/470 nm) or Dimer2/T-Sapphire (535/590 nm) was measured on an EnVision (PerkinElmer, Waltham, MA) plate reader as previously described.^32^ The FRET responses were plotted in GraphPad Prism and calculated as changes in F_CFP_/F_Citrine_ and F_T-Sapphire_/F_Dimer2_. Concentration–response curves (with mean ± SEM of EC_50_ and maximal response) were constructed on the basis of a previously described centile technique.^33^

### 2.8 Fluorescence resonance energy transfer imaging in neonatal cardiomyocytes

FRET experiments were performed 24–48 h after transfection. Cells were imaged on an inverted microscope (Olympus IX81) using an oil immersion objective (60 x 1.42 NA; Olympus). The microscope was equipped with an ORCA-AG CCD camera (C4772-80-12AG, Hamamatsu Photonics, UK) and a beam-splitter optical device (Dual-view simultaneous imaging system, DV2 mag biosystem, Photometrics, ET-04-EM). Images were acquired using custom-made software (Olympus) and processed using ImageJ. FRET changes were measured as changes in the 480/545 nm fluorescence emission intensities upon excitation at 436 nm and expressed as the per cent of 480/545 nm.

### 2.9 Fluorescence resonance energy transfer imaging in adult cardiomyocytes

Cardiomyocytes were attached to acid-washed and laminin-coated 24-mm glass cover slides in an 8-well dish transduced with the indicated biosensor for 48 h using a MOI of 1000–2000. One cover slide was placed in a watertight imaging chamber (Attofluor; Life technologies) at room temperature with buffer B (mM): MgCl_2_ (1), KCl (1.97), KH_2_PO_4_ (0.43), K_2_HPO_4_ (1.5), CaCl_2_ (1), NaCl (144), and glucose (10). One to eleven cells were imaged in parallel and were visualized consecutively every 4–5 s through a motorized digital inverted fluorescent microscope (iMIC; FEI Munich GmbH, Munich, Germany) with an air objective (10 x 0.4 NA; Olympus) or an oil immersion objective (40 x 1.3 NA; Olympus). Cells expressing the red-cGES-DE5 biosensors were consecutively excited at 422 ± 15 nm and 572 ± 15 nm (excitation of T-Sapphire and dimer2, respectively) for 20–80 ms using a monochromator (Polychrome V: FEI Munich), and emissions from T-Sapphire and dimer2 (537 ± 29 nm and 623 ± 25 nm, respectively) were separated using a Dichrotome iMIC Dual Emission Module, where a 560-nm filter separated the images from T-Sapphire and dimer2 onto a single EM-CCD camera chip (EVOLVE 512, Photometrix, Tucson, USA). Images were acquired by Live Acquisition browser (FEI), and FRET was calculated using Offline Analysis (FEI). Dimer2 emission was corrected for direct excitations at 422 nm, and spill-over of T-Sapphire emission into the 572-nm channel and changes in FRET were measured as changes in the ratios of emission from T-Sapphire and dimer2 (F_537_/F_623_). Representative experiments were run through a Savitzky–Golay filter using GraphPad 8.0.1 for Windows (GraphPad Software, San Diego, CA) for clarity. Cells expressing the cygnet2.1 biosensors were similarly excited at 436 nm and 500 nm consecutively (CFP and Citrine, respectively), and CFP and citrine emission (470 and 530 nm, respectively) was separated by a 515-nm filter onto a single EM-CCD camera. Citrine emission was corrected for direct excitations at 436 nm and spill-over of CFP emission into the 530 channel.

### 2.10 SICM–FRET

Adult rat cardiomyocytes were infected with adenovirus of the indicated biosensors, and after 48 h SICM/FRET measurements were performed as previously described^25^^,^^34^ using pipettes filled with buffer C (mM): HEPES (10); pH = 7.3, NaCl (144), KCl (5.4), MgCl_2_ (1), and CaCl_2_ (1) containing 100 µM CNP or not (control and superfusion). To ensure local stimulation of either crest or t-tubule, cells were superfused with buffer C, and release of CNP was achieved by pressure application through the pipette (276 kPa, 40 psi). FRET measurements were recorded simultaneously on a Nikon Eclipse T*i*-inverted fluorescence microscope; the biosensors were excited by a 400 nm pE-1000 light-emitting diode (CoolLED); and a DualView beam splitter (Photometrics) was used and equipped with a 565dcxr dichroic mirror and D520/30 and D630/50 emission filters. Fluorescence in individual channels was recorded by the ORCA-03G camera (Hamamatsu) using the open-source MicroManager 1.4 software. FRET ratios were corrected for the bleedthrough of the T-Sapphire into the Dimer2 channel and analysed and plotted in GraphPad Prism 8.0.1.

### 2.11 Statistics

Statistical significance was determined using GraphPad Prism with either one-way ANOVA with Tukey’s or Dunnett’s multiple comparisons test or two-way ANOVA with Sidak’s or Tukey’s multiple comparisons test or an unpaired two-sided *t*-test, where indicated. *P* < 0.05 was considered statistically significant.

### 2.12 Supplementary methods

Co-immunoprecipitation, western blot analysis, confocal microscopy, preparation of rat ventricular muscle strips, and total cGMP measurements are described in Supplementary material online.

## 3. Results

### 3.1 Generation, subcellular distribution, and characterization of FRET-based cGMP biosensors targeted to TnI and PLB

**Figure 1:**
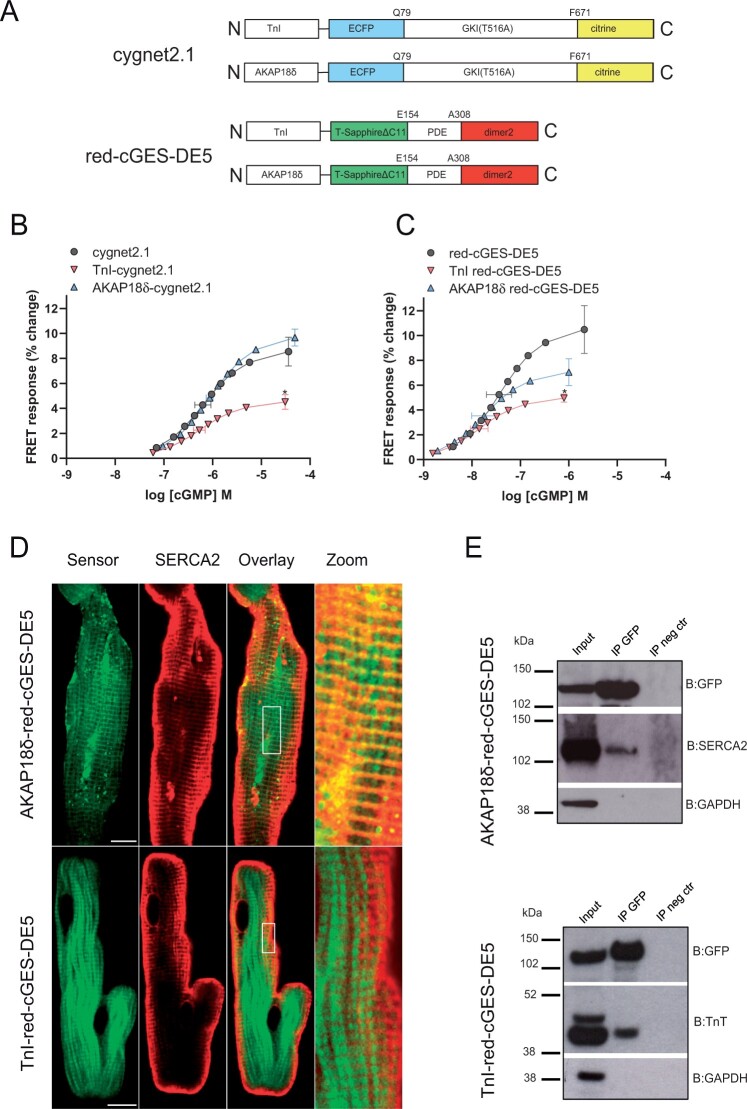
Characterization of TnI- and AKAP18δ-targeted cGMP biosensors. (*A*) Schematic domain structures of cGMP FRET-based biosensors targeted to TnI and AKAP18δ. Cygnet2.1 includes a cGMP binding domain from a truncated version of protein kinase GI (GKI) with a T516A mutation sandwiched between ECFP and citrine. Red-cGES-DE5 includes the GAF-A domain from PDE5 sandwiched between T-Sapphire and dimer2. TnI or AKAP18δ were fused to an alpha helix on the N-terminus of the biosensors. (*B, C*) HEK293T cells transfected with the indicated Cygnet 2.1 (*B*) or red-cGES-DE5 (*C*) biosensors were homogenized and incubated with increasing concentrations of cGMP. ECFP or T-Sapphire were excited at 430nm or 405nm, respectively, and the fluorescent emission (F) of ECFP/Citrine (470/515 nm) or T-Sapphire/dimer2 (590/535 nm) was measured. The FRET (F_ECFP_/F_citrine_ or F_T-Sapphire_/F_dimer2_) was then calculated. Data shown are concentration response curves with mean ± SEM of *p*EC_50_ and maximal response from four to nine experiments. *P < 0.05 vs. untargeted biosensors (one-way ANOVA with Tukey’s multiple comparisons test). (*D*) Representative confocal images of adult cardiomyocytes expressing AKAP18δ-red-cGES-DE5 or TnI-red-cGES-DE5 biosensors and immunolabelled against antibody against SERCA2 as described in ‘Methods’ section. Localization of the biosensors and SERCA2 is shown in the overlay and in the zoom. Increased contrast is applied to zoomed images. Scale bars: 10 μm. (*E*) Neonatal cardiomyocytes transfected with the indicated biosensors were lysed 24–48 h after transfection, and lysates were subjected to immunoprecipitation with anti-GFP. Direct interaction between AKAP18δ-red-cGES-DE5 biosensor and SERCA2, or TnI-red-cGES-DE5 biosensor and TnT was detected by immunoblotting with anti-SERCA2 and anti-TnT. Input is total cell lysates, and IP neg control is in the absence of anti-GFP. Shown is a representative of three independent experiments.

We have previously shown that activation of GC-A and GC-B increases total cellular cGMP to similar levels,^11^^,^^13^ yet we and others have shown that only GC-B increases phosphorylation of TnI and PLB.^10–14^^,^^17^ This is likely responsible for its lusitropic and negative inotropic effect.^17^ To directly monitor intracellular changes in cells exhibiting high and low cGMP concentrations, we selected two different FRET-based biosensors selective for cGMP, cygnet2.1 and red-cGES-DE5, that have different affinities for cGMP (EC_50_ in the sub-μM and mid-nM range, respectively).^22^^,^^23^ To determine how CNP regulates cGMP content close to TnI and PLB, we generated targeted FRET-based cGMP biosensors by fusing the N-termini of the two biosensors to TnI or AKAP18δ (that tethers PLB^24^). Fusing TnI (TnI-cygnet2.1 and TnI-red-cGES-DE5) or AKAP18δ (AKAP18δ-cygnet2.1 and AKAP18δ-red-cGES-DE5) to each biosensor (*Figure 1A*) did not modify the affinity for cGMP (*Figure 1B–C*). The potency of cGMP (EC_50_) for the untargeted, TnI- and AKAP18δ-targeted cygnet2.1 biosensors were 630, 537, and 870 nM, and the corresponding values for the untargeted, TnI- and AKAP18δ-targeted red-cGES-DE5 biosensors were 36, 14, and 17 nM, respectively. However, the two TnI-targeted biosensors displayed reduced efficacy compared to untargeted biosensors (*Figure 1B–C*). We next examined the localization of the targeted biosensors and found that AKAP18δ-red-cGES-DE5 co-localized with SERCA2 in adult cardiomyocytes (*Figure 1D*) and neonatal cardiomyocytes (Supplementary material online, *Figure S1*). SERCA2 was also co-immunoprecipitated with AKAP18δ-red-cGES-DE5 (*Figure 1E*). The TnI-red-cGES-DE5 biosensors co-immunoprecipitated with troponin T (TnT) and displayed a striated distribution in adult cardiomyocytes (*Figure 1D*), not overlapping with SERCA2 (*Figure 1D*, Supplementary material online, *Figure S1*), consistent with these not being part of the same protein complex.

### 3.2 CNP increases cGMP near TnI and PLB in neonatal cardiomyocytes

**Figure 2:**
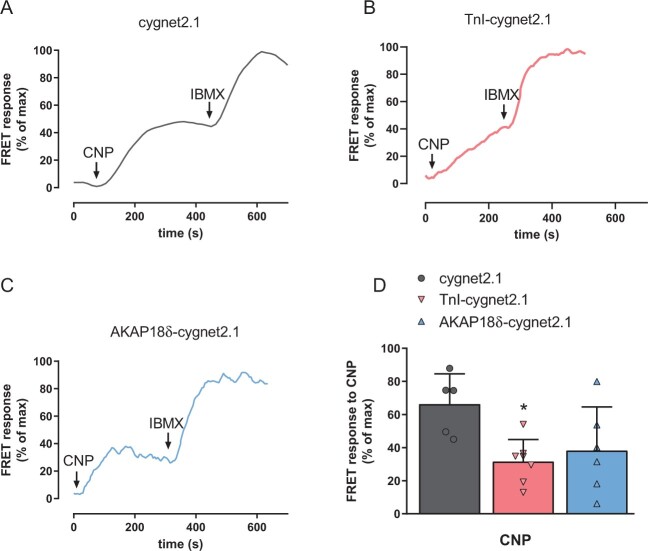
GC-B activity in neonatal cardiomyocytes. (*A*–*C*) Representative single-cell FRET recordings from neonatal cardiomyocytes expressing the indicated cygnet2.1 biosensor and stimulated with CNP (300 nM) and IBMX (100 µM) where indicated. Readouts were normalized to the maximal response (CNP + IBMX). (*D*) Quantification of FRET responses to CNP stimulation from A to C is plotted as per cent relative to maximal response. Data are mean ± SEM from five to seven cells with each biosensor from three to four independent groups of neonatal rats. *P < 0.05 vs. untargeted cygnet2.1 (one-way ANOVA with Tukey’s multiple comparisons test).

Cygnet2.1 was previously shown to be suitable to measure cGMP in neonatal rat cardiomyocytes.^26^ Here, using the untargeted, TnI- and AKAP18δ-targeted cygnet2.1 to measure cGMP changes in neonatal cardiomyocytes, we observed that CNP-stimulation increased cGMP in the cytosol and near TnI and AKAP18δ (*Figure 2A–C*). Inhibition of PDEs by IBMX (100 µM) further enhanced cGMP at all biosensors. However, this maximal change in FRET (CNP+IBMX) was significantly reduced with the TnI- and AKAP18δ-targeted biosensors compared to the untargeted biosensor (5.9 ± 1.1% and 5.5 ± 0.8% *vs.* 13.5 ± 1.3%) indicating reduced efficacy of the targeted biosensors. To control for this, we therefore report all FRET responses in per cent of maximal change observed (CNP+IBMX). However, even when taking the reduced efficacy into consideration, the CNP-stimulated change in FRET was significantly reduced (*P* = 0.023) with the TnI-targeted biosensor compared to the untargeted biosensor (*Figure 2D*), consistent with this biosensor measuring cGMP in restricted compartments. The CNP-stimulated FRET change with the AKAP18δ-targeted biosensor, on the other hand, was not significantly reduced compared to the untargeted (*P* = 0.08) or significantly different from the TnI-targeted biosensors (*P* = 0.82).

### 3.3 CNP increases cGMP near TnI and PLB in adult cardiomyocytes

**Figure 3:**
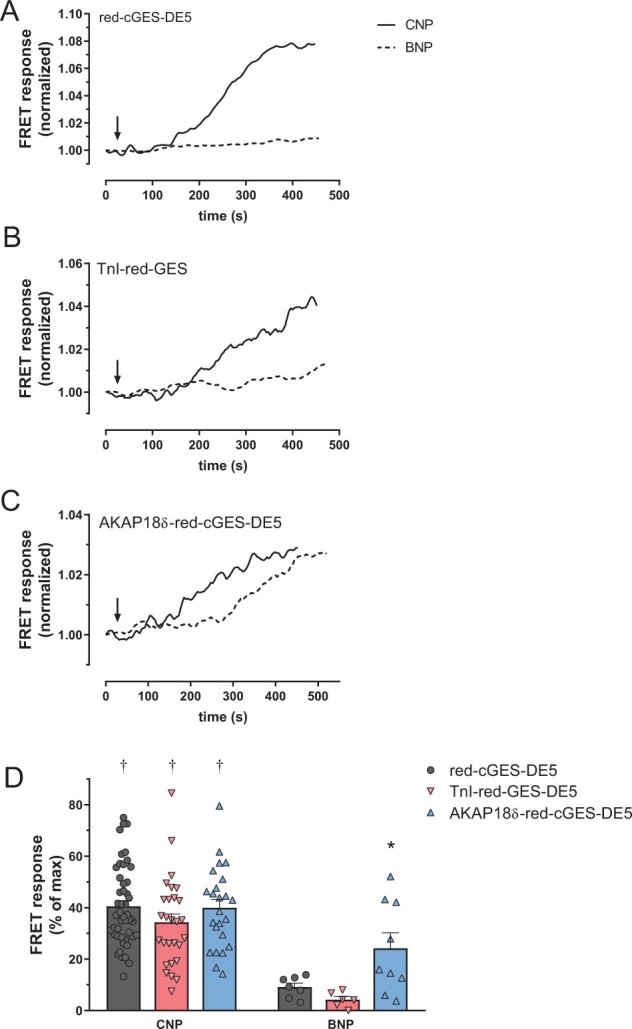
Differential GC-A and GC-B activities in adult cardiomyocytes. (*A–C*) Representative single-cell FRET responses from adult cardiomyocytes expressing the indicated red-cGES-DE5 biosensor and stimulated with CNP (300 nM) or BNP (300 nM) where indicated. Responses were normalized to signal prior to stimulation. (*D*) Quantification of FRET responses to CNP or BNP with the indicated biosensor. Responses are shown as per cent relative to maximal FRET (for CNP: subsequent addition of IBMX, for BNP: subsequent addition of IBMX and CNP) for each biosensor. Data are mean ± SEM from 6 to 48 cells from 5 to 15 animals. *P < 0.05 AKAP18δ-red-cGES-DE5 vs. TnI-red-cGES-DE5 (two-way ANOVA with Sidak’s multiple comparison test). †*P* < 0.05 CNP vs. BNP with identical biosensor (two-way ANOVA with Sidak’s multiple comparison test).

Next, we wanted to investigate the NP-induced cGMP responses in adult rat cardiomyocytes and determine whether stimulating GC-A and GC-B would increase cGMP in these compartments. Although the cygnet2.1 biosensor was efficient for measuring cGMP in neonatal cardiomyocytes (*Figure 2*), such low affinity biosensors failed to report the low cGMP concentrations in adult cardiomyocytes, both previously^14^ and in our hands (data not shown). Therefore, adult rat cardiomyocytes were infected with virus encoding the ∼20 fold more potent red-cGES-DE5 biosensor either untargeted, TnI- or AKAP18δ-targeted. CNP increased cGMP similarly across all biosensors (*Figure 3A–D*), thus indicating that stimulating GC-B increases cGMP near both TnI and PLB also in adult cardiomyocytes.

### 3.4 BNP increases cGMP near PLB in adult cardiomyocytes

We have previously shown that in cardiomyocytes from failing rat hearts, BNP increased cGMP in the bulk cytosol either significantly less than CNP,^11^ or to the same extent as CNP.^13^ Here, using cardiomyocytes from healthy adult rats, BNP increased total cellular levels of cGMP, albeit significantly less compared to CNP (Supplementary material online, *Table S1*). Using an untargeted high affinity cGMP biosensor in adult cardiomyocytes, we recently reported that BNP only increased cGMP modestly compared to CNP.^32^ We therefore examined whether BNP increases cGMP near TnI and AKAP18δ using our targeted biosensors. BNP only modestly increased cGMP with the untargeted biosensor and even less near TnI, and significantly less than CNP-stimulation (*Figure 3D*). In contrast, BNP increased cGMP significantly more in the AKAP18δ than the TnI compartment, but still significantly less than CNP (p = 0.03). This suggests that BNP increases cGMP near PLB/SERCA2, whereas CNP increases cGMP near both PLB/SERCA2 and TnI.

### 3.5 CNP increases cGMP near TnI and PLB from both t-tubules and crest

**Figure 4:**
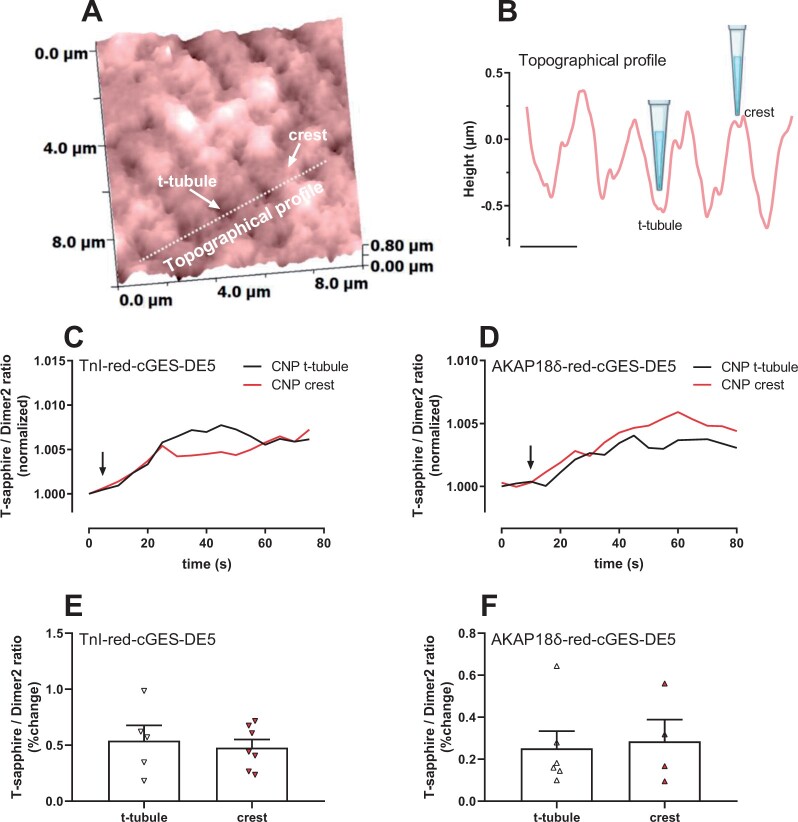
CNP stimulation of either t-tubules or cell crest increases cGMP near TnI and AKAP18δ. (*A*) Representative SICM image from a single adult cardiomyocyte expressing a targeted red-cGES-DE5 biosensor prior to local CNP stimulation on the crest of the cell and in the t-tubule as described in ‘Methods’ section. White arrows indicate t-tubule and crest structures, and the dotted line indicates the area presented in (*B*) as a topographical profile. Scale bar: 2 µm. (*C, D*) Representative SICM/FRET ratio traces of cells expressing the indicated biosensor with localized stimulation (crest/t-tubule) of CNP. FRET ratio was determined in the entire cell and normalized to that prior to stimulation. (*E, F*) Quantification of the response to CNP is shown as per cent relative to baseline. Data are mean ± SEM from four to seven cells from four animals.

Recently, different localization of GC-B was demonstrated in mouse cardiomyocytes where GC-B was evenly located across the membrane, both in t-tubules and cell crest, producing far-reaching cGMP signals.^34^ To determine whether localization of GC-B contributes to cGMP near either TnI and/or AKAP18δ in rats, we used scanning ion conductance microscopy (SICM) combined with FRET to map out the surface of cardiomyocytes and specifically stimulate with CNP inside t-tubules or on the cell crest (*Figure 4*). Both inside the t-tubules and at the cell crest, GC-B-stimulation (using a concentration of CNP that equates to 1 μM^34^) increased cGMP near TnI (FRET change t-tubules: 0.59 ± 0.11%, crest: 0.48 ± 0.07%) and AKAP18δ (FRET change t-tubules: 0.25 ± 0.08%, crest: 0.29 ± 0.10%) (*Figure 4E–F*). Stimulating with buffer in the absence of CNP did not evoke increased cGMP (Supplementary material online, *Figure S2*). This suggests that GC-B located in both t-tubule and cell crest can produce cGMP that can reach both TnI and PLB microdomains and supports such far-reaching cGMP as reported previously.^34^

### 3.6 PDE2 and PDE3 activities confine cGMP signals near TnI and AKAP18δ

**Figure 5:**
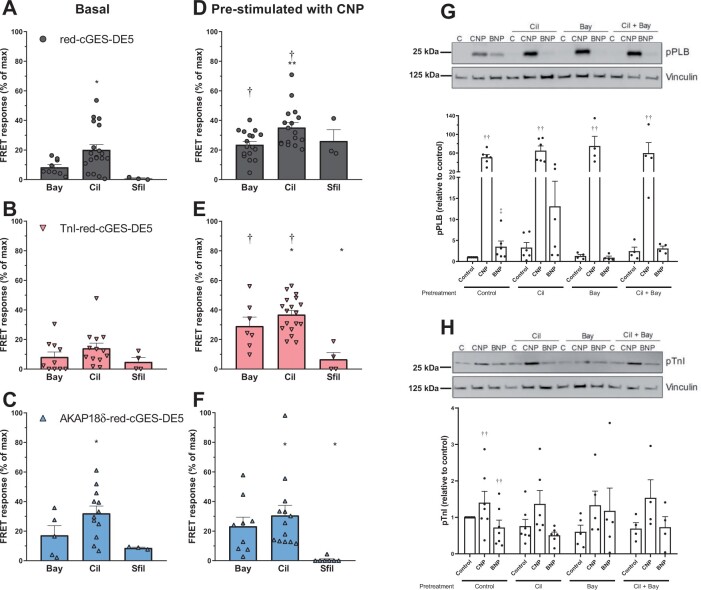
PDE2 and PDE3 regulate cGMP near TnI- and AKAP18δ-targeted red-cGES-DE5 biosensors. (*A–C*) Adult cardiomyocytes expressing the untargeted (*A*), TnI-red-cGES-DE5 (*B*), or AKAP18δ-red-cGES-DE5 (*C*) were stimulated for 5–10 min with either PDE2 inhibitor (Bay; Bay-60-7550, 50 nM), PDE3 inhibitor (Cil; cilostamide, 1 μM), or PDE5 inhibitor (Sfil; sildenafil, 100 nM). Responses were measured relative to maximal FRET obtained in that cell (subsequent addition of CNP and IBMX). Data are mean ± SEM from *n* = 3 to 19 cells from 3 to 16 animals. *P < 0.05 vs. Sfil within that group (two-way ANOVA with Tukey’s multiple comparisons test). (*D*–*F*): Adult cardiomyocytes expressing the untargeted (*D*), TnI-red-cGES-DE5 (*E*), or AKAP18δ-red-cGES-DE5 (*F*) were pre-stimulated with CNP (300 nM) for 5–10 minutes and thereafter responses to addition of the indicated PDE inhibitor were measured similar to (*A–C*). †*P* < 0.05 vs. basal PDE inhibitor response within identical biosensor. *P < 0.05 vs. Sfil within that group (two-way ANOVA with Tukey’s multiple comparisons test) and **P < 0.05 vs. Bay (two-way ANOVA with Tukey’s multiple comparisons test). (*G, H*) Adult cardiomyocytes were pretreated with the indicated PDE inhibitor for 30 minutes and then stimulated with CNP (300 nM) or BNP (300 nM) for 10 min. Cells were lysed, subjected to SDS–PAGE and blotted against *p*-Ser16-PLB (*G*) or *p*-Ser23/24-TnI (*H*), and blots were quantified relative to loading control (vinculin) and related to non-stimulated cells not pretreated with PDE inhibitor (control). Data shown are mean ± SEM from four to seven animals. ††*P* < 0.001 vs. control (two-way ANOVA with Dunnett’s multiple comparisons test). ‡*P* < 0.05 vs. control (one-sample Wilcoxon test).

Since several PDEs are responsible for tightly regulating cGMP levels in cardiomyocytes,^3^^,^^5^ we wanted to determine which cGMP PDEs displayed activity close to PLB and TnI using our biosensors. First, we assessed the regulation of basal cGMP levels. With the untargeted biosensor, inhibiting PDE2 with BAY 60-7550 (50 nM) or inhibiting PDE3 with cilostamide (1 µM) enhanced basal cGMP levels, whereas inhibiting PDE5 with sildenafil (100 nM) did not (*Figure 5A*). In the targeted biosensors, inhibiting PDE2 or PDE3 enhanced basal cGMP levels (*Figure 5B–C*). Inhibiting PDE5 had a lower effect, significant only near PLB (AKAP18δ-red-cGES-DE5 biosensor).

Next, we pre-stimulated with CNP and thereafter inhibited a PDE to determine the contribution of different PDEs to regulation of CNP-stimulated cGMP levels in the different compartments. With all three biosensors, cGMP levels were increased by inhibition of either PDE2 or PDE3, whereas inhibition of PDE5 enhanced cGMP levels only with the untargeted biosensor (Fig. 5D-F). To assess whether pre-stimulation with CNP enhanced the availability of cGMP for the different PDEs, the effects were compared with the effects on basal cGMP. The effects of PDE2 and PDE3 inhibition on CNP-stimulated cGMP levels significantly superseded the effects on basal cGMP levels only with the untargeted and TnI-targeted biosensors. Thus, the contribution of PDE2 and PDE3 to regulation of CNP-stimulated cGMP levels in the PLB compartment (AKAP18δ-targeted biosensor) is uncertain. Inhibition of PDE5, on the other hand, only increased CNP-stimulated cGMP at the untargeted biosensor (0.72 ± 0.47% of maximal FRET response in the absence *vs*. 26.1 ± 7.6% in the presence of CNP).

We next determined whether increased cGMP from BNP or CNP stimulation also increased phosphorylation of TnI and PLB. CNP stimulation alone increased phosphorylation of both PLB and TnI (*Figure 5G–H*). BNP-stimulation, on the other hand, only modestly increased PLB phosphorylation and significantly less compared to CNP stimulation (*P* < 0.001). BNP stimulation did not enhance TnI phosphorylation. These results reflect those obtained with our cGMP biosensors (*Figure 3*) by showing cGMP effects near both TnI and PLB upon CNP stimulation vs. only near PLB upon BNP stimulation. Inhibiting PDE2 or PDE3 either individually or together did not significantly enhance CNP-stimulated phosphorylation of TnI or PLB or BNP-stimulated PLB phosphorylation (*Figure 5G–H*).

### 3.7 Lusitropic and negative inotropic effects of CNP and BNP

**Figure 6:**
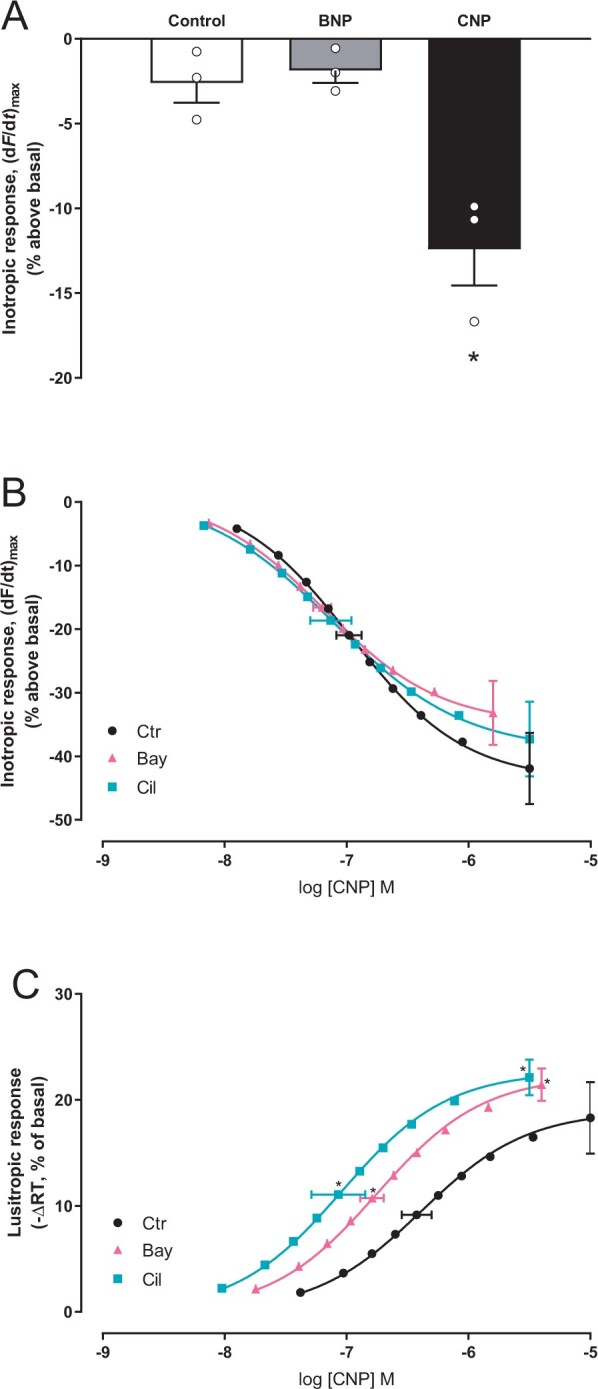
Inhibiting PDE2 or PDE3 sensitizes the CNP-induced lusitropic response. (*A*) Left ventricular muscle strips were incubated with vehicle (control) or the indicated ligand for 20–25 minutes and maximal inotropic response recorded and shown as per cent relative to that prior to stimulation (basal) (*n* = 3 strips from three animals, CNP; 100 nM, BNP; 100 nM). (*B, C*) Concentration–response curves of inotropic response (*B*) and lusitropic response (*C*) to CNP in the absence or presence of Bay (Bay60-7550, 50 nM) or Cil (cilostamide, 1 µM). Data are relative to basal (prior to CNP stimulation; *n* = 6–9). Left ventricular strips were pre-incubated with the indicated PDE inhibitor for 45 min before the first addition of CNP. Horizontal error bars depict SEM of the EC_50_, and vertical error bars are SEM of the maximal inotropic (*B*) or lusitropic (*C*) responses. *P < 0.05 vs. CNP alone (one-way ANOVA with Dunnett’s multiple comparisons test).

Next, we wanted to relate our findings to the lusitropic response (LR) and negative inotropic response (NIR) to GC-A or GC-B stimulation in rat ventricular strips. Stimulating strips with a sub-saturating concentration of CNP (100 nM) induced a NIR of 12 ± 2% (vs. 2.6 ± 1.2% in control; *P* < 0.01) (*Figure 6A*). Increasing concentrations of CNP yielded a maximal NIR of 42 ± 6% with a *p*EC_50_ of 6.9 ± 0.1 and LR (-ΔRT) of 18 ± 3% with a *p*EC_50_ of 6.4 ± 0.1 (*Figure 6B–C*). Stimulating GC-A receptors with BNP (100 nM), on the other hand, did not modify NIR relative to control (*Figure 6A*) or LR (-ΔRT of −2.2 ± 2.2 vs. 1.1 ± 0.6% in control).

We also wanted to explore whether the PDEs demonstrated to constrain the CNP-mediated cGMP with our biosensors would also affect the NIR or LR to CNP. Inhibiting PDE2 or PDE3 did not modify the maximal NIR or sensitivity to CNP (*Figure 6B*). However, inhibiting PDE2 or PDE3 both significantly enhanced and sensitized the LR to CNP (*Figure 6C*). Together, this suggests that simultaneous cGMP increase measured with both TnI- and AKAP18δ-localized biosensors closely matches the lusitropic responses observed in muscle strips.

### 3.8 CNP responses at TnI and AKAP18δ increase in heart failure

**Figure 7:**
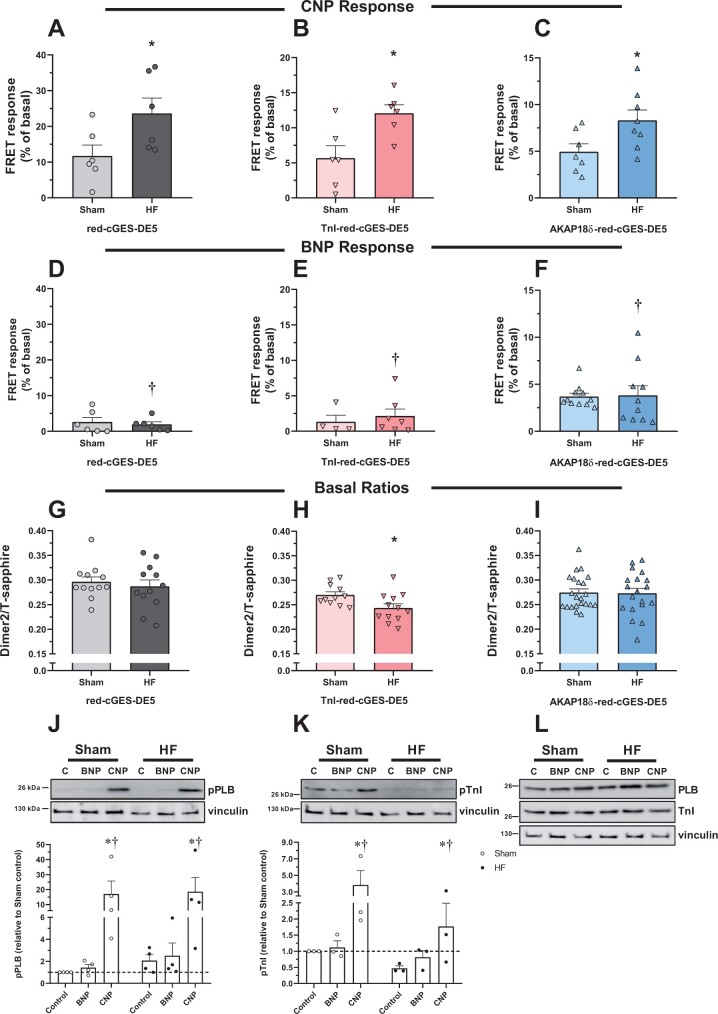
cGMP levels in Sham and heart failure adult cardiomyocytes. Rats were subjected to aortic banding (HF), or sham-operated (Sham), and 6 weeks later cardiomyocytes were isolated. Cells expressing the indicated biosensor were stimulated with either CNP (300 nM) (*A*–*C*) or BNP (300 nM) (*D*–*F*), and FRET responses were quantified relative to basal. Data are mean ± SEM from *n* = 5–13 cells from 5 to 6 animals. *P < 0.05 vs. Sham with identical biosensor (two-way ANOVA with Sidak’s multiple comparison test). †*P* < 0.005 BNP vs. CNP with identical biosensor in the same group (two-way ANOVA with Sidak’s multiple comparison test). (*G–I*): basal fluorescence intensity ratio (Dimer2/T-sapphire) in unstimulated cardiomyocytes from Sham and HF rats expressing the indicated biosensor. Data are mean ± SEM from *n* = 11 to 22 cells from five animals in each group. *P < 0.05 vs. Sham (unpaired *t*-test). (*J–L*): Cardiomyocytes from Sham or HF were stimulated with CNP (300 nM) or BNP (300 nM) for 10 min. Cells were lysed, subjected to SDS–PAGE, and blotted against *p*-Ser16-PLB (*J*) and PLB (*L*), *p*-Ser23/24-TnI (*K*) or TnI (L) and loading control (vinculin). Blots were quantified relative to loading control (vinculin) and related to non-stimulated cells in Sham. Blots are representative of three to four experiments, and data shown are mean ± SEM from three to four animals. *P < 0.05 vs. control and †*P* < 0.05 vs. BNP (two-way ANOVA with Tukey’s multiple comparison test).

We have previously shown that CNP induces a lusitropic and a negative inotropic response in heart failure (HF) that was greater than in sham-operated rats (Sham), and also that BNP does not modify lusitropic or inotropic responses in neither HF or Sham.^13^^,^^17^ In addition, the total cellular levels of cGMP upon stimulation with CNP were slightly increased in cardiomyocytes from HF.^35^ We therefore wanted to determine whether also the localized cGMP levels were altered in HF by using our targeted biosensors. Rats were subjected to aortic banding. After 6 weeks, the rats had clinical signs consistent with HF, increased heart weight, and a doubling in lung weight. In addition, echocardiography revealed increased diastolic LV diameter and posterior wall thickness and a doubling in left atrium diameter (*Table 1*). Together this indicates that the rats developed HF. In cardiomyocytes isolated from sham-operated and heart failure rats, CNP stimulation of cGMP was significantly increased in HF vs. Sham across all biosensors (*Figure 7A–C*). While activation of GC-A by BNP gave a robust cGMP response at the AKAP18δ biosensor in both Sham and HF (*Figure 7F*), there was little or no response at the untargeted and TnI biosensors (*Figure 7D–E*). The BNP-stimulated cGMP levels did not reach statistical significance from that of CNP stimulation in Sham at the untargeted (*P* = 0.06), TnI (*P* = 0.09) and AKAP18δ (*P* = 0.5) biosensors, but were significantly lower than CNP in HF across all biosensors (*P* < 0.005) (*Figure 7D-F*).

**Table 1:** Characteristics of Sham and AB rats

Animal characteristics	Sham	AB
Body weight (BW) (g)	422 ± 5	378 ± 11*
Heart weight (g)	1.93 ± 0.11	3.33 ± 0.34*
Lung weight (g)	1.73 ± 0.06	3.46 ± 0.16*
HW/BW (g/kg)	4.1 ± 0.3	7.9 ± 0.5*
M-mode		
IVSd (mm)	1.3 ± 0.0	1.8 ± 0.1*
PWd (mm)	1.4 ± 0.1	2.4 ± 0.1*
LVDd (mm)	8.1 ± 0.2	9.0 ± 0.2*
LVDs (mm)	4.8 ± 0.1	6.1 ± 0.5
FS (%)	40 ± 1	33 ± 5
LAD (mm)	3.4 ± 0.2	7.3 ± 0.4*
Doppler recordings		
Peak mitral flow (m/s)	1.13 ± 0.06	1.57 ± 0.06*
Mitral deceleration (mm/s^2^)	3.5 ± 0.5	7.5 ± 0.6*
Peak RVOT (m/s)	0.9 ± 0.0	0.9 ± 0.1
Heart rate (bpm)	366 ± 11	308 ± 6*
Maximal tissue velocity (mm/s)	53 ± 2	35 ± 1*
Minimal tissue velocity (mm/s)	53 ± 4	45 ± 5

BW, body weight; FS, fractional shortening; HW, heart weight; IVSd, intraventricular septum in diastole; LAD, left atrium diameter; LVDd/s, left ventricular diameter in diastole or systole; LW, lung weight; PWd, posterior wall in diastole; RVOT, right ventricular outlet tract.

In Sham and AB rats. *P < 0.05 vs. Sham (unpaired, two-sided *t*-test). *n* = 6 in both groups.

The increased cGMP seen in HF could be due to lower basal cGMP levels, and we therefore determined the basal FRET ratio (Dimer2/T-sapphire) with all biosensors. There was no change in basal FRET at the untargeted and AKAP18δ biosensors, but at the TnI-red-cGES-DE5, we observed a slightly decreased basal FRET (*Figure 7G–I*). Since the red-cGES-DE5 biosensor decreases FRET in response to increased cGMP,^23^ this suggests that there is a higher basal cGMP level in HF with the TnI biosensor.

We then determined whether phosphorylation of PLB and TnI was modified in HF. CNP stimulation significantly increased both TnI and PLB phosphorylation in both Sham and HF (*Figure 7J–K*). However, both basal (control) and CNP-stimulated TnI phosphorylation in HF were almost half of that in Sham. This was not due to reduced TnI expression in HF, as both TnI and PLB were unaltered in HF (*Figure 7L*).

## 4. Discussion

We developed and validated targeted FRET-based biosensors to monitor cGMP levels in the vicinity of TnI and PLB. We used these biosensors to show that CNP stimulation increases cGMP near TnI and PLB in both neonatal and adult rat cardiomyocytes, whereas BNP only increases cGMP close to PLB. This may explain why stimulation with CNP, but not BNP, can regulate contractility. Further, we report that GC-B in either t-tubule or cell crest can generate this cGMP and that PDE2 and PDE3 are involved in regulating localized CNP-stimulated cGMP levels and lusitropic responses. In addition, we found that the localized cGMP from GC-B is enhanced in HF, and that localized cGMP from GC-A is preserved in HF.

### 4.1 Real-time detection of cGMP inside cardiomyocytes near TnI and PLB

Cygnet2.1 is an efficient biosensor for detecting cGMP in neonatal rat cardiomyocytes, shown herein and previously.^26^ However, as demonstrated here and previously,^14^ it was not sufficiently sensitive to measure cGMP changes in adult rat cardiomyocytes. This could either be due to lower concentrations of cGMP surrounding the biosensors in adult vs. neonatal cardiomyocytes, or due to lower GC-B receptor density. Therefore, biosensors with higher affinity for cGMP, such as the red-cGES-DE5 or *Pf*PKG biosensors,^32^ are needed to report cGMP levels in adult cardiomyocytes.

Targeted FRET-based biosensors are often hampered with reduced FRET efficiency due to the targeting domain being closely attached to one of the fluorophores and likely influencing the relative orientation and/or distance between the FRET pairs.^36–38^ Despite our efforts to minimize such interactions, by spacing the fluorophore and targeting domain with an alpha helical linker, our targeted biosensors still displayed reduced efficiency *in vitro* (TnI biosensors) and in cardiomyocytes (TnI and AKAP18δ biosensors). To maintain FRET efficiency in targeted biosensors, a better strategy would be to use FRET-based cGMP biosensors with an N-terminus devoid of fluorophores where a targeting domain can be placed, such as for the CUTie cAMP biosensor.^38^ Unfortunately, such cGMP biosensors with high affinity for cGMP do not exist so far.^39^

The targeting domains used for our cGMP biosensors are identical to those used in the targeted CUTie cAMP biosensors,^38^ and subcellular localization of our cGMP biosensors was confirmed through co-immunoprecipitation and confocal microscopy (*Figure 1*). Even though both targeted biosensors gave similar CNP responses, the pattern of BNP stimulation and PDE inhibition was different between untargeted and targeted biosensors (*Figures 2–5*), further suggesting differential subcellular localization of the targeted biosensors. The untargeted biosensors would represent the bulk cytosolic cGMP generated after CNP stimulation, of which all is not compartmented around TnI and PLB.

### 4.2 Concerted cGMP increase near both TnI and AKAP18δ is required for lusitropic and negative inotropic responses

The most striking finding in the present work was that CNP stimulation in adult cardiomyocytes increased cGMP similarly in both cytosol and near AKAP18δ and TnI, which was in sharp contrast to BNP stimulation that increased cGMP more near AKAP18δ compared to the cytosol and TnI. However, the BNP-stimulated cGMP near AKAP18δ was notably lower than after CNP stimulation in adult cardiomyocytes. Phosphorylation of PLB and TnI followed a similar pattern, where CNP stimulation increased phosphorylation of both, whereas BNP stimulation only increased PLB phosphorylation, also significantly lower than with CNP stimulation. These findings vary somewhat from data from mouse cardiomyocytes and rats with heart failure, where GC-A and GC-B stimulation increased cGMP similarly, but only CNP increased PLB phosphorylation.^13^^,^^14^^,^^34^ In the present study, HF did not alter the effect of BNP stimulation, but enhanced the cGMP increase upon CNP stimulation across all biosensors, corresponding to the increased total cellular cGMP levels observed with a different rat HF model.^35^

We have previously demonstrated that a lusitropic and a negative inotropic response to CNP required phosphorylation of at least PLB.^17^ In the present work, we show that BNP (at least partially) increases phosphorylation of PLB, but not TnI (*Figure 5*). However, this does not translate into a lusitropic or negative inotropic response to BNP (*Figure 6*). Although the CNP-stimulated TnI phosphorylation was reduced in HF without a concomitant loss in total TnI content, CNP still increased phosphorylation by the same percentage as in Sham due to lower basal TnI phosphorylation (*Figure 7*). Since stimulating GC-B in HF also causes a lusitropic and negative inotropic response,^11–13^^,^^17^ this may indicate that the increase in phosphorylated TnI is sufficient to preserve these responses.

The BNP-stimulated cGMP near AKAP18δ and also PLB phosphorylation were lower than with CNP (*Figures 3, 5, and 7*). This could be due to either i) that the lower amount of cGMP from GC-A only activates a subset of PKG and hence achieves a lower degree of PLB phosphorylation, ii) different membranal localization of these receptors, or iii) that GC-A-generated cGMP only has access to a limited pool of PLB. At least less cGMP from stimulation of GC-A is likely, since BNP-stimulation yielded lower total cellular cGMP content than CNP both herein and in mouse cardiomyocytes.^14^ However, in heart failure cardiomyocytes, we previously reported similar total cellular cGMP response to both BNP- and CNP-stimulation.^13^ This may indicate that different subcellular localization of GC-A and GC-B could mediate different cellular responses and could account for the lower BNP-stimulated cGMP near PLB and lower PLB phosphorylation seen in this study. In terms of localization of receptors at the membrane, we demonstrate that at least GC-B located either in t-tubules or at the cell crest can increase cGMP near AKAP18δ or TnI (*Figure 4*). The lusitropic and negative inotropic responses obtained with CNP would then be a result of GC-B activation on either t-tubules or cell crest that increases both TnI and PLB phosphorylation. Although there is evidence of different pools of PLB,^40^ whether GC-A only has access to a limited pool of these, whereas GC-B has access to a larger pool and also the importance of such different pools of PLB in regulating NIR and LR, is unknown. Nevertheless, our present data corroborate our previous finding that both increased TnI and PLB phosphorylation are needed for CNP to initiate NIR and LR. We proposed a mechanism for this, where PLB phosphorylation enhances Ca^2+^ sequestration into the SR, leading to faster relaxation and, in the absence of L-type calcium channel and ryanodine receptor phosphorylation (as opposed to the situation with cAMP from β-adrenergic stimulation), depletion of Ca^2+^ available for contraction, and TnI phosphorylation increases the rate of Ca^2+^ dissociation from TnC.^17^ Based on our present data, both are required to observe the NIR and LR. GC-A activation, on the other hand, only leads to a partial phosphorylation of PLB (and not TnI), resulting in no alteration of lusitropic or inotropic responses.

Increasing afterload in animal models with cardiomyocyte-specific deletion of GC-B resulted in increased passive tension and diastolic dysfunction compared to WT animals.^16^ Since the CNP-mediated phosphorylation of PLB and TnI and subsequent faster relaxation (lusitropic response) is present in both normal^18^ (*Figure 6*) and HF^17^ animals, it would be of interest to explore whether these mechanisms contribute to maintain diastolic function in models with increasing afterload.

### 4.3 PDE2 and PDE3 confine the cGMP responses near TnI and PLB

We have previously shown that CNP-stimulation enhances the cAMP-mediated inotropic response through cGMP-mediated inhibition of PDE3,^11^^,^^20^^,^^35^ and that PDE3 inhibition sensitizes the potency of CNP-induced NIR and positive LR in normal and failing hearts.^11^^,^^12^^,^^35^ Here, we demonstrate in normal rat hearts that both PDE2 and PDE3 are important regulators of cGMP concentrations near TnI and PLB (*Figure 5*) and restrain the CNP-mediated LR (*Figure 6*). Our previous report in HF rats detected an increase in CNP-stimulated global levels of cGMP by inhibiting PDE2 and not PDE3,^13^ whereas others have reported in rat and mouse cardiomyocytes that CNP-induced cGMP is regulated by PDE3 and not PDE2.^14^^,^^34^^,^^41^ Using our targeted biosensors, we demonstrate an increased cGMP after inhibiting either PDE2 or PDE3. This suggests that the local PDE activity important in regulating these compartments is not necessarily detected when measuring global cGMP levels. Inhibiting PDEs with IBMX always yielded higher CNP-stimulated levels of cGMP, suggesting that other PDEs than PDE2 or PDE3 could also have roles in regulating these compartments. Since PDE9 has been shown to regulate some aspects of cGMP signalling in cardiomyocytes,^4^ and this is insensitive to IBMX, we cannot rule out the contribution of PDE9 to regulate cGMP from either GC-A or GC-B.

Although inhibiting either PDE2 or PDE3 increased the maximal CNP-mediated LR (*Figure 6*), it did not significantly enhance phosphorylation of PLB or TnI (*Figure 5*). Such discrepancy was also observed in HF.^12^ This could either be due to lower sensitivity in phosphorylation measurements or other factors in addition to PLB and TnI phosphorylation that could be important in regulating LR.

The basal cGMP levels were highly constrained by PDE3 in the AKAP18δ compartment (*Figure 5C*). This is in line with studies investigating cAMP compartmentation near PLB,^37^ and studies directly addressing PDE3 activity near SR,^42^^,^^43^ and suggests that local pools of cAMP and cGMP exist near PLB/AKAP18δ in unstimulated cardiomyocytes, but these are constitutively dampened by the presence of PDE3.

### 4.4 GC-B located both in t-tubules and on cell crest

Cardiomyocyte plasma membrane structure is highly complex. Some of us have previously reported in mouse cardiomyocytes that GC-A is localized strictly to t-tubules, whereas GC-B is evenly dispersed both in t-tubules and on the cell crest.^34^ In this report, we also find that GC-B is localized both in t-tubules and on the cell crest and these receptors increase cGMP around TnI and AKAP18δ (*Figure 4*). Since TnI and AKAP18δ display different intracellular localization (myofilament and SR, respectively), this suggests that cGMP propagates similarly from t-tubules to both myofilaments and SR. Alternatively, GC-B receptors are not homogenously distributed in t-tubules, and our method cannot separate between GC-B located on different areas of the t-tubules.

## 5. Conclusion

To conclude, CNP increases cGMP near both TnI and PLB and causes a lusitropic response, which is regulated by PDE2 and PDE3, as well as a negative inotropic response. Using targeted FRET biosensors, we report that receptors in both cell crest and t-tubules are responsible for these effects. Stimulation with BNP, on the other hand, increases cGMP near PLB which alone does not regulate inotropic and lusitropic responses. In heart failure, CNP stimulation of cGMP levels is enhanced.

## Supplementary material

Supplementary material is available at *Cardiovascular Research* online.

## Authors’ contributions

O.M., G.C., V.O.N., M.Z., F.O.L., and K.W.A. designed the research. O.M., G.C., A.F., D.A., N.C.S., S.M., A.O.M., M.A., J.M.A., L.R.M., and K.W.A. performed the research. All authors analysed the data. O.M., F.O.L., and K.W.A. wrote the paper. All authors revised and approved the manuscript.

## Funding

This work was supported by the South-Eastern Norway Regional Health Authority (grants 2011025 and 2019051), the Norwegian Council on Cardiovascular Diseases, The Research Council of Norway (grants 205167 and 303490), The Anders Jahre Foundation for the Promotion of Science, The Family Blix Foundation, The Simon Fougner Hartmann Family Foundation, grants from the University of Oslo, and grants from British Heart Foundation (Programme Grant RG/17/6/32944) to M.Z.

**Conflict of interest:**None declared.

## Supplementary Material

cvab167_Supplementary_Data

## Notes

Translational Perspective Although best known as heart failure biomarkers, natriuretic peptides (ANP, BNP, and CNP) are important signalling molecules in the heart and other organs through increasing cyclic GMP (cGMP). Treatment preventing their degradation improves heart failure prognosis. To better understand their cardiac signalling, we employed fluorescent cGMP biosensors targeted to troponin I and phospholamban and found that BNP and CNP increase cGMP differently around these proteins in both normal and failing cardiomyocytes. This may explain the different effects of BNP and CNP on cardiac contractility and relaxation, with possible implications for understanding and treatment of heart failure.
