# Supplementary material for: CNP regulates cardiac contractility and increases cGMP near both SERCA and TnI: difference from BNP visualized by targeted cGMP biosensors
Source: Cardiovasc Res. 2021 May 10;118(6):1506–19. doi: 10.1093/cvr/cvab167 (PMC9074987; doi:10.1093/cvr/cvab167)
Supplement: cvab167_Supplementary_Data [file cvab167_supplementary_data.pdf]

Supplementary Material

Manfra *et al.* *"CNP regulates cardiac contractility and increases cGMP near both SERCA and TnI – difference from BNP visualized by targeted cGMP biosensors"*

Supplementary methods

S1.1 Co-immuniorecipitation

Neonatal cardiomyocytes were transfected with the indicated plasmid or infected with the indicated adenovirus, washed after 24-48 h with ADS buffer (mM): NaCl (106), Hepes (20), NaH_2PO_4 (0.8), KCl (5.3), $MgSO_4$ (0.4), glucose (5) and lysed in RIPA buffer (mM): NaCl (150), Tris-HCl (50) pH 7.5, NP-40 1 %, aprotinin 0.1 %, PEFA-block (1), leupeptin 10 μg/ml, β-glycerolphosphate (10) for 6 min. Then, cells were collected and rotated for 10 min at 4°C and protein expression was measured with Bradford protein assay. Proteins (750 μg) were added to 30 μl of GFP-TRAP beads or 15 μl of control beads (ChromoTek, Planegg-Martinsried, Germany) in a final volume of 500 μl and rotated for 2 h at 4°C. Beads were then washed 3-4 times with 500 μl RIPA buffer and subsequently denatured with a mixture of NuPage LDS Sample Buffer and NuPage sample reducing agent (Life Technologies) and RIPA buffer. Proteins were separated on a Gel NuPage Gradient 4-12 % gel and transferred onto a nitrocellulose membrane. Immunodetection was performed with the following primary antibodies: Serca2 (c-20) goat polyclonal; troponin T-c (c-19) goat polyclonal; GAPDH (H-12) mouse; GFP (B-2) mouse antibodies (Santa Cruz Bio technologies), and the following secondary antibodies: antibody anti-goat HRP (Santa Cruz Bio technologies), anti-mouse HRP (Promega).

S1.2 Western blot analysis

Total cell lysates were prepared in Urea lysis buffer: urea 8 M, thiourea 2 M, Tris-HCl 0.05 M, pH 6.8 at 22°C and aliquots of approximately 10 μg were electrophoresed on 4-15 % precast

TGX gels (Bio-Rad Laboratories, Hercules, CA). Proteins were transferred to nitrocellulose membranes. The blots were blocked in Tris-buffered saline containing 0.1 % Tween 20 (TTBS) with 5 % non-fat dry milk, cut for visualization of vinculin and thereafter incubated with the indicated primary antibody in TTBS with 5 % non-fat dry milk or 5 % BSA overnight at 4°C. The blots were washed twice in TTBS before incubation with secondary antibody for 1 h at room temperature. LumiGLO (KPL, Gaithersburg, MA) was used for visualisation of the blots. Densitometric analyses were done using Labworks Software (UVP, Cambridge, UK). When quantifying western blots, the data were normalized using the signal strength of vinculin.

S1.3 Confocal microscopy.

24-48h after transfection or transduction with the indicated biosensor, neonatal and adult cardiomyocytes were fixed in 4 % paraformaldehyde, permeabilized in 0.2 % Triton-X100 and incubated with Serca2 (c-20) goat antibody over-night at 4°C. Serca2 was then visualized with Alexa Fluor 568- or Alexa Flour 647-conjugated anti goat antibodies (Life technologies) and mounted in DakoCytomation Fluorescent Mounting Medium (DakoCytomation, Carpinteria, CA, USA). Confocal images for neonatal cardiomyocytes were acquired with Olympus Fluoview FV1000 (Olympus Corporation, Tokyo, Japan) on an inverted IX81 motorized microscope. Excitation at 488 nm was used to reveal the cygnet2.1 biosensors and excitations at 559 nm or 635 nm were used to reveal the secondary antibodies. Confocal images for red-cGES-DE5 in adult cardiomyocytes were analyzed using an Olympus FV1000/BX61 with 488, 405, 543 and 633 nm lasers and an oil immersion objective (60 x 1.35 NA). Multi-labeled images were acquired sequentially and single-image TIF-files were exported to ImageJ (U. S. National Institutes of Health, Bethesda) for creating an overlay image.

S1.4 Preparation of rat ventricular muscle strips.

Left ventricular muscle strips were prepared, mounted in organ baths (31 °C) as previously described[1] with Ca^{2+} concentration of 1.8 mM, equilibrated and field-stimulated with 10–15 mA (20 % above beating threshold) at 1 Hz. Contraction-relaxation cycles (CRC) were recorded and analyzed as previously described[1] with respect to maximal development of force $(dF/dt)^{max}$, time to peak force (TPF), time to 80 % relaxation (TR^{80}) and relaxation time ($\Delta RT = TR^{80}$-TPF). Negative inotropic responses (NIRs) were expressed as changes of $(dF/dt)_{max}$ in percent of basal levels (before addition of agonist). Lusitropic responses (LRs) were expressed as increases of -ΔRT in percent of basal level. Blockers of α_1-adrenoceptors (prazosin 1 μM), β-adrenoceptors (timolol 1 μM), muscarinic cholinergic receptors (atropine 1 μM), lidocain (20 μM) and ascorbid acid (100 μM) were added 90 min prior to the indicated agonist. These drugs did not influence the basal CRC characteristics or electrical stimulation threshold (not shown). Basal developed force in muscle strips was 4.9±0.5mN (n=6). Where indicated, the indicated PDE inhibitor was added 45 min prior to CNP. Cumulative doses of CNP were added to the organ bath to obtain concentration-response relationships. Concentration-response curves were constructed by estimating centiles (EC_{10}-EC_{100}) for the receptor-selective effects for each experiment and calculating the corresponding means and the horizontal positioning expressed as –log EC_{50}, as previously described.[2]

S1.5 Total cGMP measurements.

Isolated ventricular cardiomyocytes were stimulated with BNP (100 nM) or CNP (100 nM) for 10 min. Cyclic GMP levels were measured using a cGMP enzyme immunoassay kit (Cayman Chemical Company, Ann Arbor, MI, USA) as previously described.[3]

References

1. Sjaastad I, Schiander I, Sjetnan A, Qvigstad E, Bokenes J, Sandnes D, Osnes JB, Sejersted OM, Skomedal T. Increased contribution of α_1- vs. β-adrenoceptor-mediated inotropic response in rats with congestive heart failure. *Acta Physiol Scand* 2003;177:449-458.
2. Skomedal T, Borthne K, Aass H, Geiran O, Osnes JB. Comparison between alpha-1 adrenoceptor-mediated and beta adrenoceptor- mediated inotropic components elicited by norepinephrine in failing human ventricular muscle. *J Pharmacol Exp Ther* 1997;280:721-729.
3. Moltzau LR, Aronsen JM, Meier S, Nguyen CH, Hougen K, Orstavik O, Sjaastad I, Christensen G, Skomedal T, Osnes JB, Levy FO, Qvigstad E. SERCA2 activity is involved in the CNP-mediated functional responses in failing rat myocardium. *Br J Pharmacol* 2013;170:366-379.

Supplementary Table S1. BNP and CNP increase total cellular cyclic GMP

Adult cardiomyocytes were stimulated for 10 min with either BNP (100 nM) or CNP (100 nM), lysed and cGMP levels were determined as described in Materials and Methods. Shown are means±SEM from 4 independent experiments. * $p<0.05$ *vs*. BNP.

**Stimulation**	**cGMP (fmol/mg protein)**
Basal	40 ± 27
BNP	391 ± 128
CNP	1617 ± 340 *

Supplementary Figure S1. Subcellular localization of the TnI- and AKAP18δ-targeted biosensors in neonatal cardiomyocytes.

Confocal images of neonatal cardiomyocytes co-expressing the indicated targeted cGMP biosensors (TnI-cygnet2.1, AKAP18δ-cygnet2.1, TnI-red cGES-DE5 or AKAP18δ-red cGES-DE5) and immunolabeled for SERCA2, as described in Materials and Methods. The localization of the biosensor and SERCA is shown in the overlay and in the zoom. Scale bars=10μm.

Supplementary Figure S2. Buffer control experiments of t-tubules or cell crest.

A) Representative SICM/FRET ratio traces of cells expressing the indicated biosensor with localized stimulation (crest/t-tubule) of buffer in the absence of CNP. FRET ratio was determined in the entire cell and normalized to that prior to stimulation. **B)** Quantification of the responses in A are shown as percent relative to baseline. Data are mean±SEM from 2-4 cells.

Supplementary Figure S1

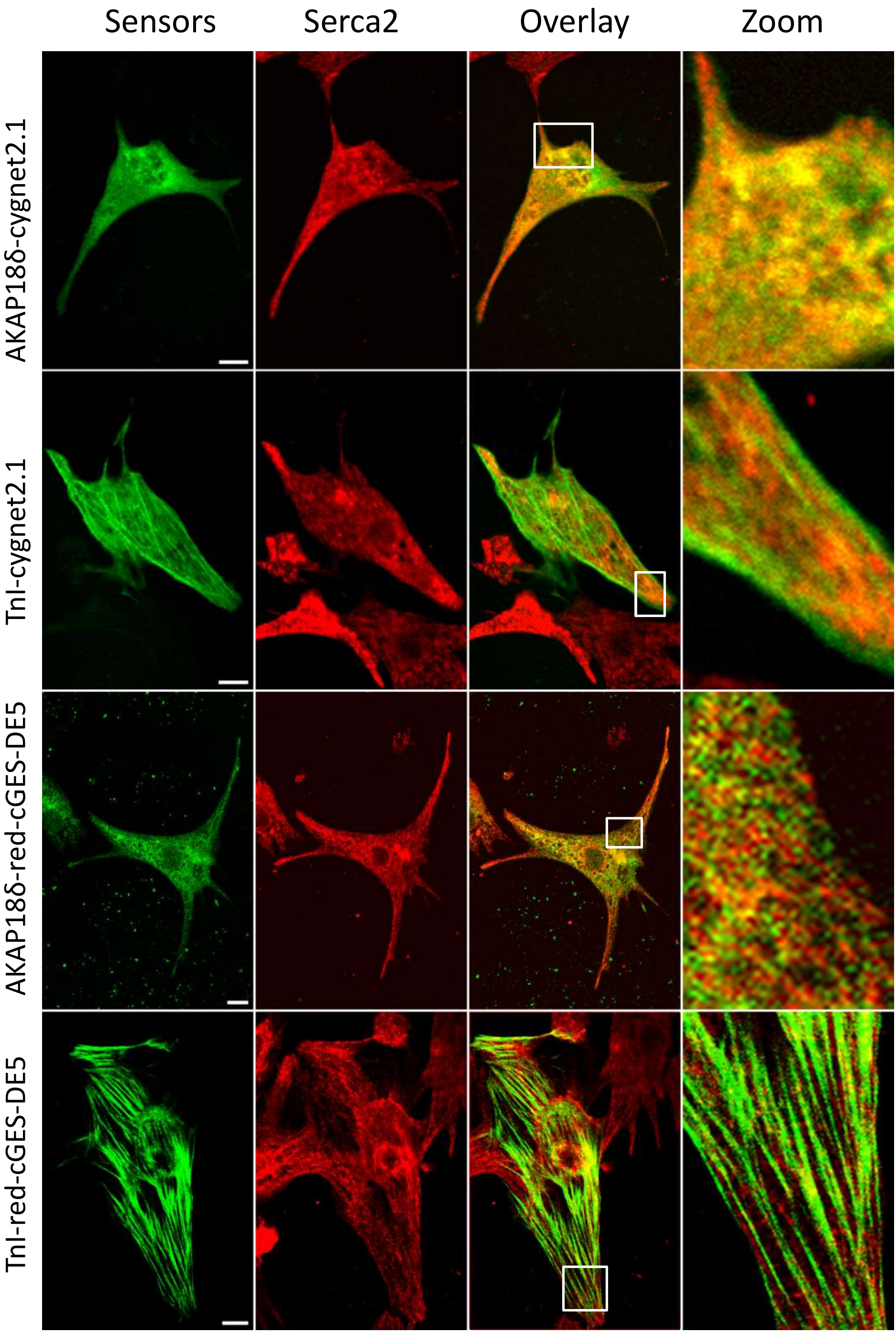

Supplementary Figure S2

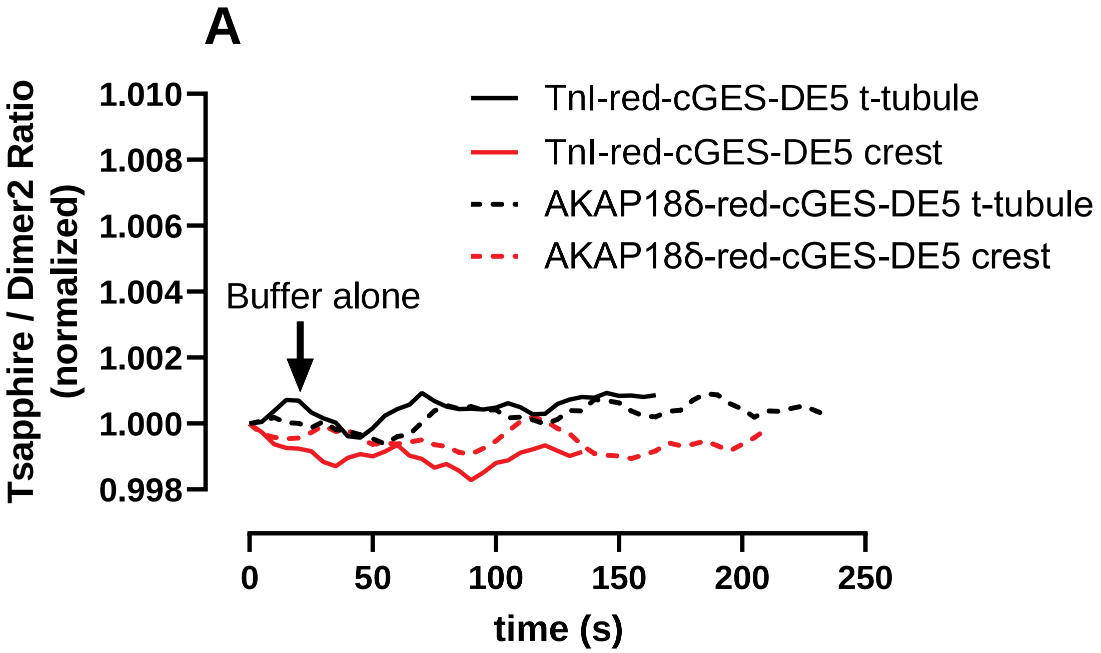

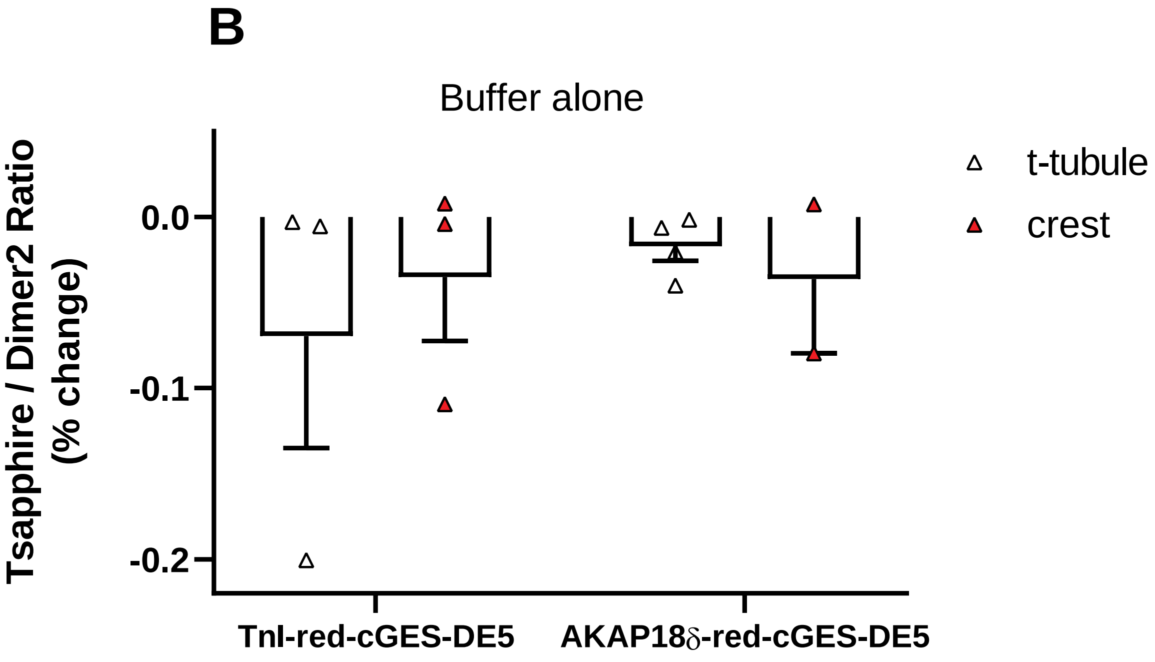

Supplementary Figure S3. Full blots in Figure 1E

AKAP18δ-red-cGES-DE5

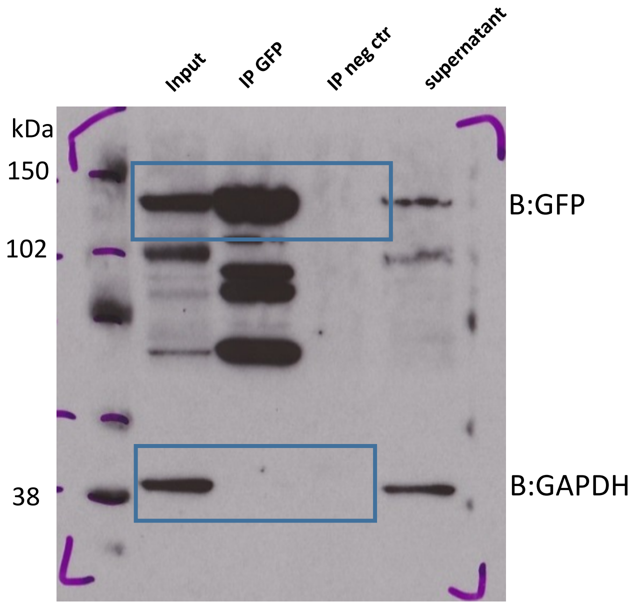

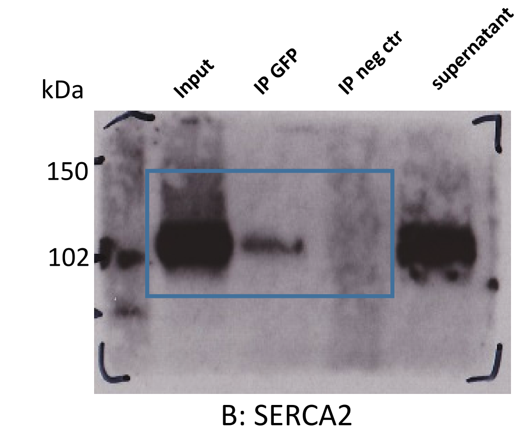

TnI-red-cGES-DE5

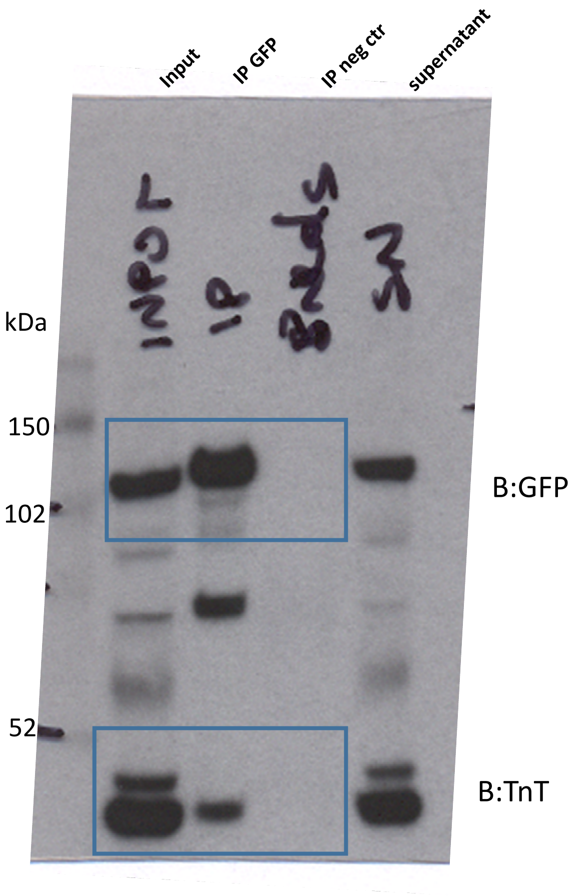

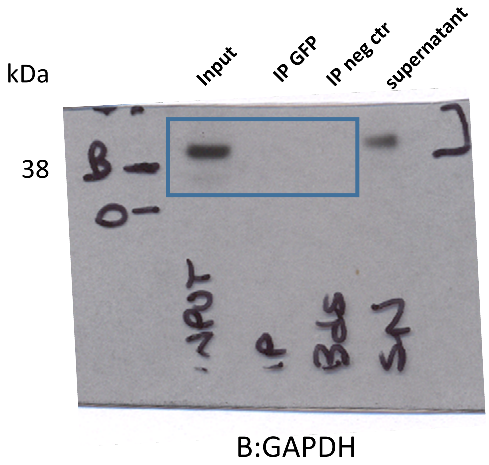

Supplementary Figure S4. Full blots in Figure 5

Figure 5G

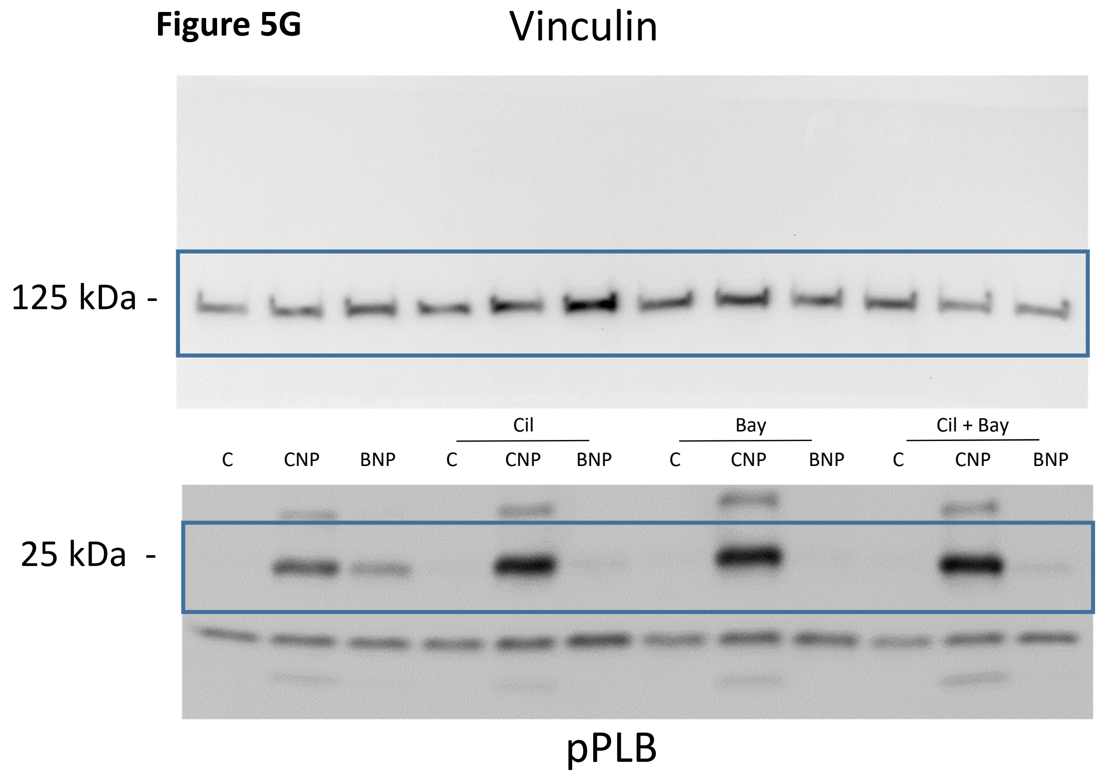

Figure 5H

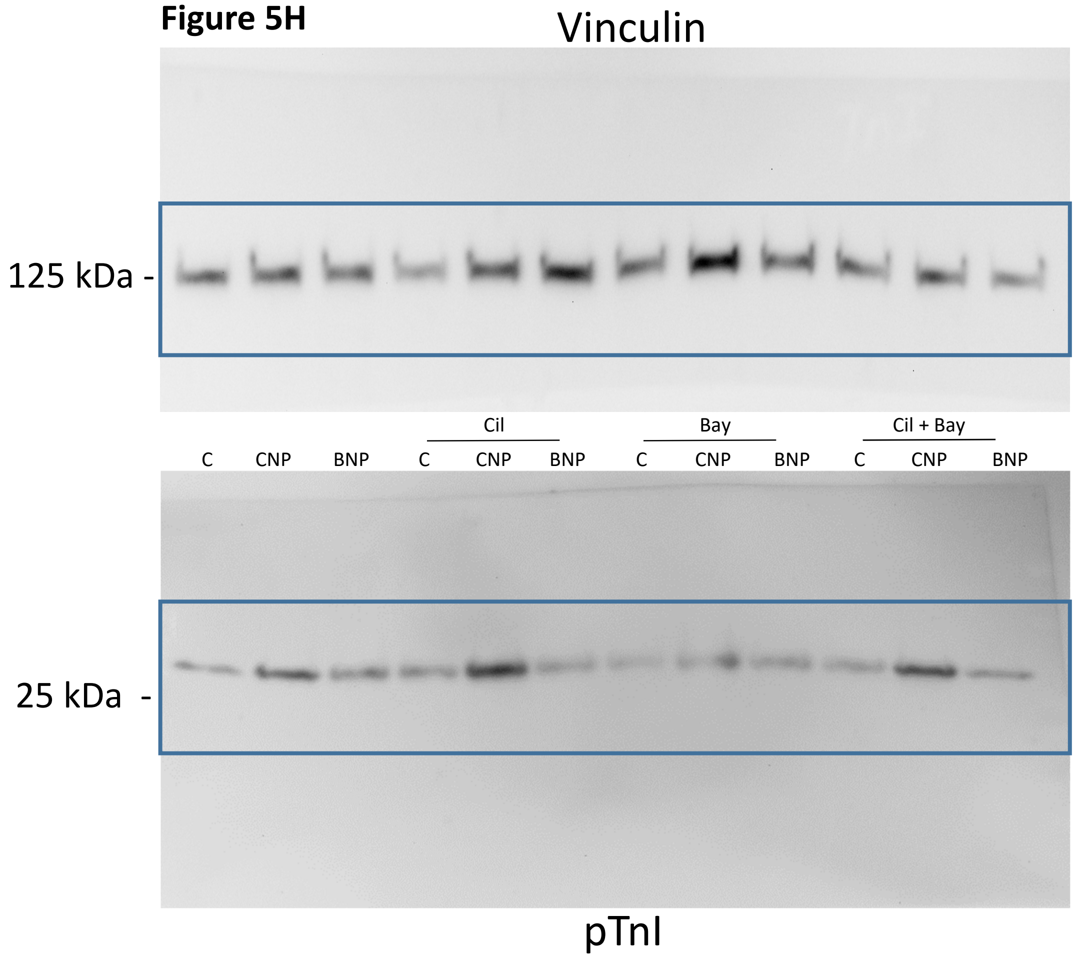

Supplementary Figure S5. Full blots in Figure 7

Figure 7J

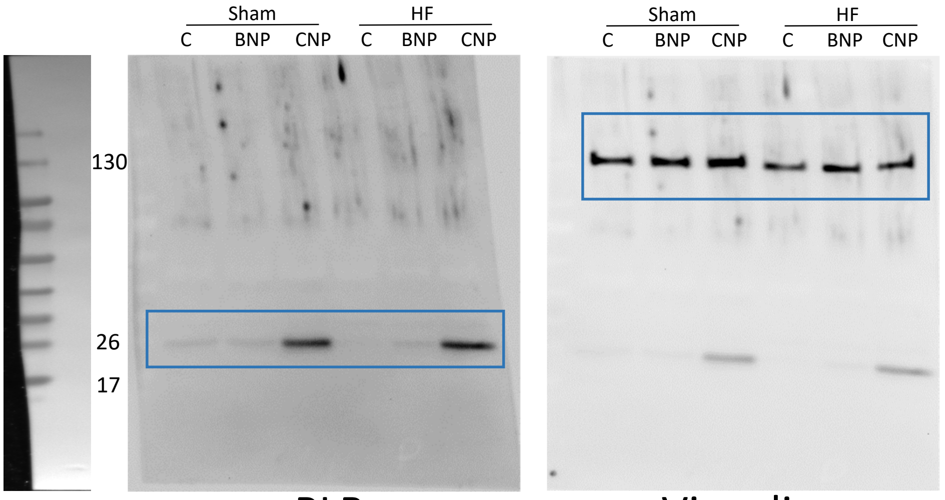

pPLB Vinculin

Figure 7K

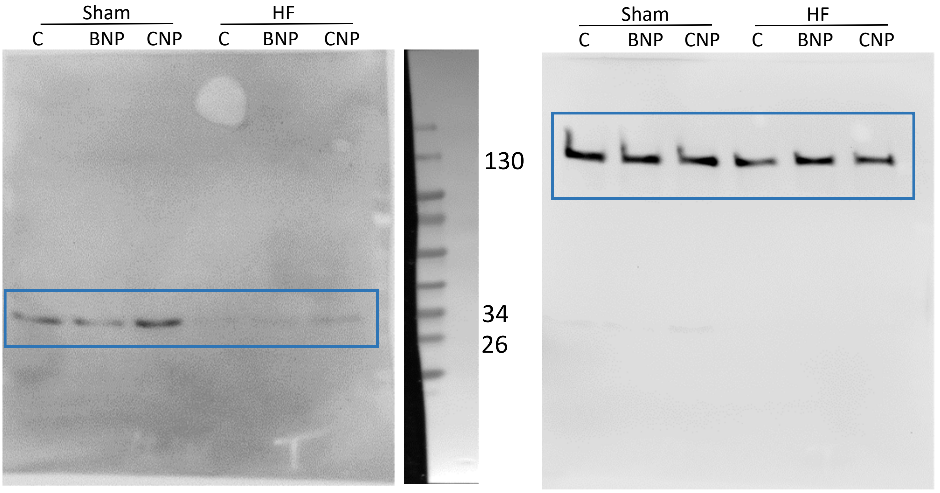

pTnI Vinculin

Figure 7L

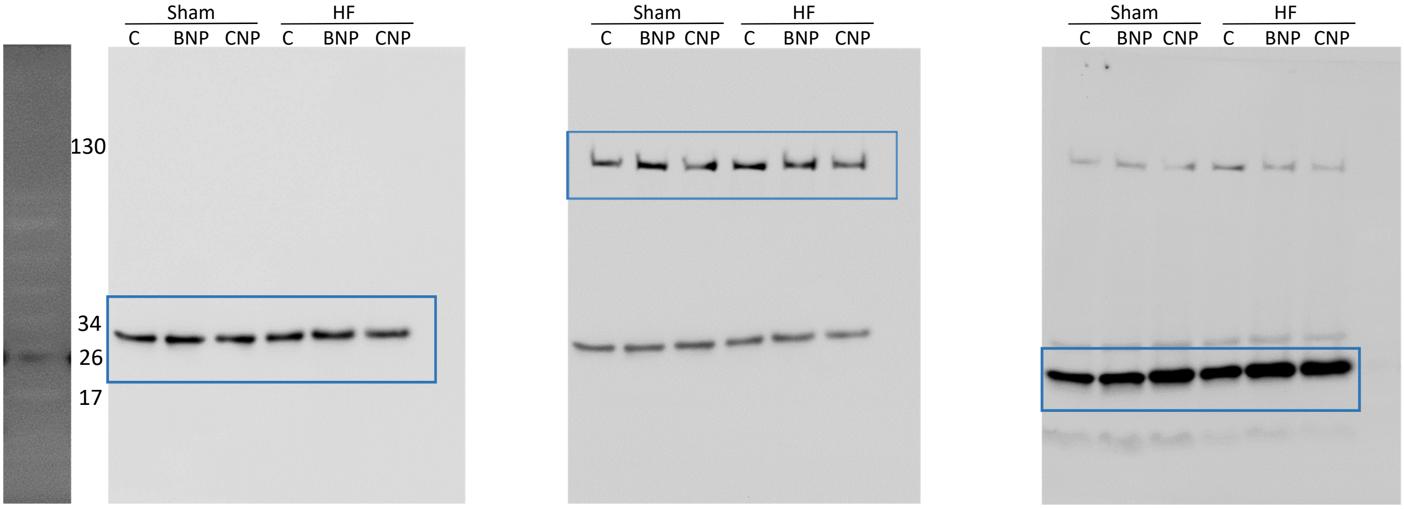

TnI= incubation 1 Vinculin= incubation 2 PLB= incubation 3
